# Development of a computerized intervention to improve health literacy in older Hispanics with type 2 diabetes using a pharmacist supervised comprehensive medication management

**DOI:** 10.1371/journal.pone.0263264

**Published:** 2022-02-09

**Authors:** Joshua Caballero, Robin J. Jacobs, Raymond L. Ownby

**Affiliations:** 1 Department of Clinical and Administrative Pharmacy, College of Pharmacy, University of Georgia, Athens, Georgia, United States of America; 2 Departments of Health Informatics, Nutrition, Medical Education & Research, Kiran C. Patel College of Osteopathic Medicine, Nova Southeastern University, Fort Lauderdale, Florida, United States of America; 3 Department of Psychiatry and Behavioral Medicine, Kiran C. Patel College of Osteopathic Medicine, Nova Southeastern University, Fort Lauderdale, Florida, United States of America; GITAM College of Pharmacy: GITAM Institute of Pharmacy, INDIA

## Abstract

**Objective:**

The primary objective was to develop a computerized culturally adapted health literacy intervention for older Hispanics with type 2 diabetes (T2D). Secondary objectives were to assess the usability and acceptability of the intervention by older Hispanics with T2D and clinical pharmacists providing comprehensive medication management (CMM).

**Materials and methods:**

The study occurred in three phases. During phase I, an integration approach (i.e., quantitative assessments, qualitative interviews) was used to develop the intervention and ensure cultural suitability. In phase II, the intervention was translated to Spanish and modified based on data obtained in phase I. During phase III, the intervention was tested for usability/acceptability.

**Results:**

Thirty participants (25 older Hispanics with T2D, 5 clinical pharmacists) were included in the study. Five major themes emerged from qualitative interviews and were included in the intervention: 1) financial considerations, 2) polypharmacy, 3) social/family support, 4) access to medication/information, and 5) loneliness/sadness. Participants felt the computerized intervention developed was easy to use, culturally appropriate, and relevant to their needs. Pharmacists agreed the computerized intervention streamlined patient counseling, offered a tailored approach when conducting CMM, and could save them time.

**Conclusion:**

The ability to offer individualized patient counseling based on information gathered from the computerized intervention allows for precision counseling. Future studies are needed to determine the effectiveness of the developed computerized intervention on adherence and health outcomes.

## Introduction

Health literacy (HL) is a crucial skill needed by patients to maintain adherence to treatment and achieve positive health outcomes. Approximately 25% of Americans have difficulties understanding healthcare information [1]. Racial and ethnic minorities have lower levels of HL than non-Hispanic whites; 41% of Hispanics have some of the lowest levels of HL [1]. Additionally, older persons from racial and ethnic minorities are more frequently affected by chronic diseases [1].

Nonadherence has been reported in patients with T2D [2–4]. A meta-analysis found adherence rates for diabetes treatments of approximately 68%, discontinuation rates in controlled trials of 32%, and twelve-month persistence rate of 56% [4]. Nonadherence is a combination of multiple factors. As a result, a tailored comprehensive intervention that identifies modifiable factors may assist providers to identify key areas of need (e.g., improving understanding of illness or benefits of medications, recommending/directing care). Information tailored to patient interests, needs, and characteristics has a greater impact on behavior than simply providing the same information without tailoring [5, 6].

Researchers have argued for the usefulness of computer-delivered interventions in communicating health information [7–9]. The primary method of provider-patient communication about medications has been brief and hurried oral communications. Data show physicians may not sufficiently promote adherence due to lack of time or by failing to utilize effective communication formats for the patient (e.g., appropriate reading level) [10]. Studies suggest patient retention of information in this context is poor, often less than 50% [11]. Therefore, novel computerized interventions have been proposed to improve HL with the aim of increasing adherence and health outcomes especially in underserved communities [12].

There is an increasing role for pharmacists in improving adherence in patients with T2D. Studies show pharmacists can improve adherence and HbA1c concentrations through comprehensive medication management (CMM) [13]. CMM is part of the patient care process that guarantees each medication is individually assessed for appropriateness, effectiveness, and safety for the individualized patient [14]. A key element of the CMM includes an interactive, face-to-face medication review and consultation on a patient’s medications by a pharmacist or other qualified provider. The main goal of the tailored CMM consults is to assess medication therapy to improve adherence and outcomes. The improvement of health outcomes includes educating the patient regarding their disease state, counseling on medications, and discussing the importance of adherence. CMM includes discussing intended goals and outcomes of treatment while verifying the patient understands, agrees with, and actively participates in treatment. However, studies on the benefits of CMM (e.g., HbA1c, blood glucose levels, quality of life) have been inconclusive because of limitations or failure to demonstrate efficacy [4, 15]. A key limitation in these studies is their focus on adherence as an isolated problem. There are additional factors that impact adherence which may include financial burden, mental health, and accessibility [16, 17]. Additionally, the information provided may not be dovetailed to the patient’s specific needs, rather following a narrative script with ubiquitous pre-printed materials requiring an extended period of time.

Given that factors affecting adherence and health outcomes are multi-factorial, an intervention that is itself multifactorial and based on an assessment of modifiable factors is more likely to be effective. As a result, a computerized intervention that provides basic information on T2D and prompts the patient to answer questions regarding the material may allow the pharmacist to streamline the medication review process of CMM. The intervention should also have queries on other health factors impacting adherence, and reasons for non-adherence that will assist the provider to address or refer other services. The results of the intervention may allow for *precision counseling* since the pharmacist would have the information needed to systematically address individual patient concerns. The primary objective of this study was thus to develop a computerized culturally specific HL teaching intervention for Hispanic older adults with T2D. Secondary objectives were to assess the usability and acceptability of the intervention by Hispanic older adults with T2D and pharmacists providing CMM.

## Materials and methods

A three-phase study was approved by Nova Southeastern University’s Institutional Review Board (2017-408-NSU). To participate in the study, older Hispanic participants with T2D needed to be 50 years of age or older, have a hemoglobin A1c > 7%, have been prescribed diabetes medications, and able to communicate in Spanish. Key informants included clinical pharmacists who completed their doctorates in pharmacy, residency trained, and/or had previous experience in serving the Hispanic population and T2D. Participants who had severe cognitive or psychiatric complications interfering with the ability to understand key elements of the study were excluded. Participants meeting inclusion criteria were consented and provided $40 for their involvement in the study.

During phase I, an integration approach using quantitative assessments and qualitative interviews was used to develop the intervention and ensure its cultural suitability [18]. The quantitative assessment was conducted with the researcher using a pen and paper format, input into REDCap database management software, and ultimately transferred to SPSS statistical software for final analysis (Version 26; Armonk, NY: IBM) [19].

Qualitative methodologies are particularly important for the development of conceptual frameworks that focus on the individual, developmental, and sociocultural contexts in which behavior occurs [20]. A better understanding of these can serve as an essential guide to intervention development. This approach also helps us understand some of the cultural issues within a group sharing a similar, potentially life-altering experience [21]. The semi-structured qualitative interviews were digitally recorded, transcribed, coded, and analyzed. Data saturation was reached at the 15th participant. Using an iterative process, qualitative data from the interviews were hand-coded by the researchers, compared, and organized into themes to elucidate the findings from the quantitative survey. An inductive thematic analysis (ITA) was conducted to allow for the patterns, themes, and categories of analysis to emerge and reveal data about participants’ views, opinions, knowledge, experiences, or values that may not be captured in quantitative assessments. Braun and Clarke’s guidelines for ITA were used to identify themes related to health literacy regarding T2D and its management, capturing individual understanding and allowing an in-depth analysis of the data [22]. We also used the framework recommended in the literature used to satisfy the criteria for trustworthiness, which includes credibility (validity); transferability (generalizability); dependability; and confirmability [23–25]. Topics in the semi-structured qualitative interview (developed by the researchers) addressed knowledge of T2D and medications, medication management attitudes and behavior, and access to services.

Transcripts from the recordings combined with observational notes from the researchers were used in an iterative process. The hand-coded data were organized by the researchers into major themes that emerged. Themes were cross-checked by the researchers for accuracy. Using an iterative process, the coded data were then organized into final major themes. Two authors reviewed transcripts and field notes of the interviews to identify all relevant ideas. Their notations were compared and discussed. Descriptive codes based on patterns within the qualitative interview data were collated with primary attention to identify salient themes across the quantitative data. The process was repeated until there was agreement on the ideas and concepts. Quantitative measures used in this study included demographics, Marin Acculturation Scale (MAS), and Language Experience and Proficiency Questionnaire (LEAPQ) [26, 27]. Acculturation and language experience/proficiency were assessed using the MAS and LEAPQ, respectively. All quantitative analyses were conducted using SPSS.®

During phase II, the intervention was edited and translated from English into Spanish. It was also adapted for cultural relevance based on the integration of the quantitative and qualitative interviews in phase I and the clinical and cultural expertise of the researchers. The translation and adaptation efforts utilized a process of successive review and revision of the working drafts of the intervention, as suggested by Devieux et al. and conducted in the field by other researchers [28–30]. The intervention content includes questions previously developed and used in our FLIGHT/VIDAS research and addressed general knowledge related to T2D and medication-specific knowledge [31]. Some of the information used (e.g., diabetes pathophysiology) was previously piloted to explore relationships between patient HL and mental health (e.g., cognition, depression, anxiety) [32, 33]. After each segment on the educational slides, an item appears testing the user’s knowledge. The computerized intervention also includes information addressing barriers for adherence including injectable administration as reported in the literature [34]. For example, if a user states he or she is taking insulin, the program automatically directs the user to a section discussing insulin and its proper use. Also included are items that test knowledge gained. Some items ask the user if he or she would like further instructions on how to administer the medication (e.g., insulin).

During phase III, the intervention was tested for usability and acceptability similarly to previously published methodology [28, 35, 36]. Initially, five users were observed interacting with the computer intervention and asked to “think out loud” so their cognitive processes could be tracked and areas of difficulty identified and corrected. Afterward, based on feedback from the first five users, the intervention was revised and retested with five different participants. After the testing usability and acceptability with ten participants, we explored the usability of the intervention to incorporate into CMMs with five clinical pharmacists, all of whom had experience with our study population and were thus able to check the concordance and trustworthiness of the intervention.

Questions asked during the interviews with the clinical pharmacists included, “Overall, the computer program would be easy for patients to use,” and “Learning to operate a touch screen computer for the intervention would be easy for a patient” using a Likert-type response set “strongly agree” to “strongly disagree.” Additional statements pertaining to integration with conducting CMM were included such as “The intervention can save me time when conducting CMM”, “The intervention can allow me to systematically assess the needs of my patient”, and “The intervention is easy to use in my practice when performing CMM.” The pharmacists provided feedback regarding changing some content to more accurately represent typical life situations. They also suggested changes to make the intervention easier to use in CMM. The ultimate goals of phase III were to have an intervention which was not just user friendly to the patient but would also be user friendly for pharmacists to optimize their effectiveness when completing CMM.

## Results

In phase I, the sample (*N* = 15) consisted of older Hispanics with formal education. Table 1 reports the summary statistics of the MAS and T2D variables. The participants’ ages ranged from 55–84 years (*M* = 68.9 years; *SD* = 8.66). About half were women (*n* = 8; 53.3%). Data from the LEAP-Q showed all participants reported Spanish was their native and dominant language and learned English as second language. The sample was heterogeneous in terms of place of origin: Cuba, Ecuador, Costa Rica, Puerto Rico, Argentina, and Mexico (none were born in the United States) and immigrated as early as 1961 and as recently as 2018. All participants identified racially as “white” and ethnically as “Hispanic/Latinx”. The mean score for the MAS was 1.76 (*SD* = .51), suggesting a strong preference for using Spanish in most contexts. The MAS had good internal reliability with this sample (alpha = .918). For the single item not included in the scale, *How would you describe yourself*?, 54.3% (*n* = 8) reported “very Latinx/Hispanic” and 46.7% (*n* = 7) reported “more Latinx/Hispanic than American.”

**Table 1:** Phase I: Summary statistics of the MAS and T2D variables (N = 15).

Variable	Mean	Std. Dev.	Min.	Max.
Marin Acculturation Scale (MAS)*	1.76	.511	1	3
T2D diagnosis (self-reported) (in years)	8.43	9.12	3	40
HbA1c levels	7.48	.262	7.1	7.9

*Lower scores = lower acculturation; scores ranged from 1 = only Spanish, 2 = more Spanish than English, 3 = both equally, 4 = English better than Spanish, and 5 = only English. T2D (type 2 diabetes).

Years since T2D diagnosis (self-reported) ranged from 3 to 40 years (*M* = 8.43; *SD* = 9.124). Self-reported HbA1c levels ranged from 7.1 to 7.9 (*M* = 7.48; *SD* = .262). Participants reported having comorbid illnesses such as depression, hypertension, rheumatoid arthritis, asthma, and lipid disorder. Medications for glycemic control most reported by participants included metformin 67% (*n* = 10), insulin 67% (*n* = 10), glyburide 27% (*n* = 4), and sitagliptin 13% (*n* = 2). Other medications included pioglitazone, glipizide, dapagliflozin, and liraglutide.

Five major themes emerged from qualitative interviews conducted in phase I: 1) financial considerations, 2) polypharmacy, 3) social and family support, 4) access to medication/information, and 5) loneliness and sadness. Many participants discussed the high cost of insulin, even with government assistance, rendering them unable to pay (e.g., insurance deductible, copay or coinsurance). Some participants reported obtaining insulin from outside the U.S. (e.g., trips to Ecuador) where it is substantially less expensive, albeit in limited quantities and with unknown quality.

One participant reported when asked if he/she/they used less of a medication due to lack of money to pay for it:

“I always pay later since I take a lot of medications… Then, they sent me a letter, but they didn’t have metformin, so I couldn’t pay for it that day. Well, I fought, and they gave me the medications, but it was very expensive.”

Participants reported polypharmacy issues and discussed problems with having to remember to take multiple medications and adhere to a complicated medication regime. For example, one participant stated:

“Yes, sometimes, because there are so many medicines that I have to take every day, each day, two or three times a day, that I get tired…others require food, and then, others are before bedtime. All of them are different and very complicated.”

Another participant commented:

“There are so many medications. Sometimes, I forget. I have all of my diabetes medications, the insulin. I also have one for high blood pressure.’”

Outside of the immediate family, participants reported they do not always tell friends they are on medications for T2D. Their responses suggested they feel other people do not ask or do not care, so they do not mention it. For some of the men, due to certain Hispanic cultural traits regarding men are to remain strong and serve as the provider, it may be uncomfortable to rely on others [37]. One male participant, when asked “Who helps you [remember to take medications],” responded:

“No, no, no, no. My wife is with me, but I control myself. I won’t say that she’s the one who tells me, this is like this, and this is like that. No, I control myself…” another respondent noted “No, absolutely no one helps me to take my medications. I always have to remind myself of what time I have to take my medications. Because honestly, I have no one to help me. The only ones who worry about me are my children, but they’re not here.” Conversely, participants who reported high levels of social support, particularly from family members, reported high levels of medication adherence. When asked who is supportive of taking insulin, one participant said “Here, all of my children, grandchildren, neighbors…my husband, my grandchildren, no, it’s very, very good. I always take it.”

The themes regarding access to reliable health information in the Spanish language emerged. Due to this, they may be forced to search for information from possibly non-reliable sources found on the Internet. For example, when asked where they go when they need to look for information about a new medication one participant replied:

“I go to Google and look it up to see what it causes me, whether there are any changes in the body, why I would use it. I check all of that.”

The participants who reported using online sources of information indicated they used only Google and YouTube. One participant reported having to look to YouTube to learn how to inject insulin as the provider did not sufficiently explain how to. Conversely, another participant, when asked if they ever go to the Internet to look for information, responded “No, I don’t have the internet nor a cell phone.”

Lastly, emotional distress emerged as a theme related to barriers to medication adherence. Participants reported worrying about relatives (e.g., grandchildren) and loneliness as cause for impediments to medication adherence. For example, one woman stated she stopped taking medication for T2D due to sadness and loneliness:

“Yes, I do feel sad because I’m a woman who is alone…honestly, yes, there are times when I feel lonely.”

While sadness and loneliness were discussed, depression itself did not emerge as a major consideration as a barrier to medication adherence in this sample. However, 33% (n = 5) were taking antidepressants.

During phase II, the intervention was revised from data collected. Some revisions included information related to the importance of medication adherence and lifestyle modifications (e.g., diet, exercise, smoking, vaccinations). Additionally, questions were added regarding lifestyle modification, medication administration, family/social support, depression items, and barriers to medication access.

During phase III, grammatical edits were conducted and content was reduced (e.g., information on incretin deleted). The participants during phase III (*N* = 10) felt each module of the intervention would take approximately 20 minutes (range: 15–25 minutes) and having the information broken into three modules was sufficient. The clinical pharmacists (*N* = 5) agreed including an item (i.e., visual analog scale) directly asking about medication adherence would be important to add. Based on data from phase I on mood (e.g., sadness, loneliness) and their clinical experience, pharmacists felt a depression screening tool is beneficial and thus the Patient Health Questionnaire-2 question (PHQ-2) was added [38]. Participants felt the computer program was easy to use (n = 4 agree; n = 6 strongly agree) and learning to operate the touch screen computer was also easy (n = 4 agree; n = 6 strongly agree). All pharmacists also agreed the intervention can save them time when conducting CMM, allowed them to systematically assess the needs of their patient, and the intervention would be easy to use in practice when performing CMM.

## Discussion

A computerized intervention was developed and culturally adapted for pharmacists to use to improve HL in Hispanics with T2D. We identified five major themes in the qualitative analysis and were able to develop an intervention to address them. Users found the intervention easy to use and clinical pharmacists agreed that it could streamline the medication review process of CMM. A finding in this study is that medication costs, particularly for insulin, may impact adherence. This is in line with the rising cost of insulin in the U.S. and out of pocket expenses for patients under Medicare Part D plans [39]. Additionally, a recent study evaluating approximately 165,000 adult patients (<65 years of age) with diabetes reported 41% had financial difficulties from medical bills, and approximately 50% showed cost-related medication non-adherence [40]. Assisting patients to find ways to better afford their medications may thus be an important strategy to improve adherence.

Another key element identified was polypharmacy. At this time, the primary method of provider-patient communication about medications has been brief oral communications during sometimes hurried medical consultations. Studies have suggested patient retention of information in this context is poor, often less than 50% [11, 41, 42]. In this study, clinical pharmacists found the computer application easy to use, time effective, and may provide a systematic method to assess their patients when providing CMM. Section 10328 of the Affordable Care Act amended section 1860D-4(c)(2)(ii) of the Act requires prescription drug plan sponsors (per Part D Plans) to offer an annual comprehensive medication review [43]. Therefore, the ability to use an interactive computerized intervention may assist pharmacists and other health care providers in offering a dovetailed process focusing on the patient specific needs and improving care. However, future studies are needed to determine if this computerized tailored intervention can improve retention, adherence, and health outcomes (e.g., decreased HbA1c).

Computerized interventions that can actively engage patients and extract individualized assessments of HL can help pharmacists access current and relevant information to provide *precision counseling*. At this time, there is no clear definition for *precision counseling*. Currently, according to the National Institute of Health, precision medicine is the approach for disease treatment and prevention that uses individual variability in genes, environment, and lifestyle for each person [44, 45]. This approach allows prescribers to predict which treatment and prevention strategy may produce the best outcome in a specific patient and is in contrast to the one-size-fits-all approach. Patient education and counseling traditionally follow a systematic approach often using pre-printed information or script. For example, according to ASHP Guidelines on pharmacist conducted patient education and counseling, there are sixteen points that may need to be addressed for each medication prescribed to a patient [46]. These guidelines are congruent with the requirements provided by the Medicare Part D medication therapy management program standardized format [43]. If the average patient who is being educated is taking multiple medications, this process can become cumbersome, time consuming, and prevent a patient from processing crucial information. Similarly to precision medicine, *precision counseling* uses all available information that may impact adherence (e.g., HL, cost, mental health) and allows a pharmacist or healthcare provider to develop an optimal plan to improve medication adherence and health outcomes based on the responses to queries answered by the patient while using an intervention such as this one. Therefore, *precision counseling* can be defined as the approach for disease treatment and prevention that takes into account social factors, environmental stressors, medication side effects, and lifestyle impacting medication adherence for each individual person. Similar to a blood test to determine genetics, pharmacodynamic, and pharmacokinetic variables to drive precision medicine, a computerized intervention such as this one would allow for using *precision counseling* to improve health outcomes.

Social family support was another theme that emerged from our study. The data are not clear how the role of family and social support plays in adherence and improved health outcomes in adults with T2D [47]. Regardless, that is not always the case. Some studies show patients may be criticized or scolded when receiving social support [48]. Additionally, competing demands (e.g., family/friends eating differently than patient with diabetes) can negatively impact a patient’s adherence to a healthier lifestyle. However, when the quality of the family-patient relationship is positive, the outcomes improve [49]. For example, data show when there are positive influences and family dimensions, adherence may increase by 27%. Therefore, it is important for healthcare providers to assess not just if a caregiver exists in the patient’s life, but how encouraging they are. Strong family social support is described as warm, accepting, and close and thus future interventions may focus on evaluating this relationship (i.e., strong vs. poor social/family support).

The methods our older Hispanic participants with T2D used to access medication/information were intriguing. While there is useful content on the internet for medication related information, using YouTube may not offer the most accurate or reliable information and can be time consuming. YouTube contains anecdotal evidence and misleading information that may contradict professional, reliable information, suggesting a need to develop interventions that assist patients to access medical and scientific facts related to their illness and treatment. The current computerized intervention was developed using guidelines and professional resources that provides the patient with trustworthy information and could improve their HL. The ability for healthcare providers to direct their patients to useful, correct, and interactive information may provide for better adherence and outcomes. For example, information tailored to patient interests, needs, and characteristics has a greater impact on behavior than simply providing the same information without tailoring [5, 6]. Tailored information interventions may have an impact on behavior similar to motivational interviewing [3]. Specific aspects of tailoring may be more effective than others, such as those enhancing perceived personal relevance of material that may increase processing of the information presented as specified by the Elaboration Likelihood Model [6, 2]. Multimedia presentation of material may also increase learning and retention and possibly helpful in various health conditions and populations [50–52]. For example, a single blind randomization study using web-based education among older adults 65 years of age or older demonstrated improvement in HL drug utilization [53]. Several authors have also argued for the potential usefulness and cost-effectiveness of computer-delivered multimedia interventions in communicating health information [7–9]. Multimedia instruction with graphics and narration may be useful strategies for helping patients acquire the knowledge and skills needed to effectively maintain adherence over time. One study showed supplementing verbal presentations with pictures had a positive effect on behavior in patients with low HL [54]. As a result, HL interventions that are computerized, use multi-media, and are engaging have shown positive outcomes and should be at the forefront of future research efforts.

Additionally, factors that affect mental health (i.e., loneliness, sadness) may impact medication adherence and appeared as a theme. A third of our participants were taking antidepressants and while depression itself was not mentioned, the terms used (e.g., loneliness, sadness) may reflect features of depression. This is supported by data showing almost 50% of Hispanics use terms that differ from Western labels for depression [55]. Depression affects almost 20% of patients with T2D and has been associated with poor adherence and outcomes [56–59]. Rates of depression in Hispanics with T2D are even higher. A recent study in Hispanics in South Florida showed 80% of participants met criteria for depression, while 80% and 83% of participants reported poor self-efficacy and non-adherence, respectively [60]. In other Hispanic populations, patients with depression were more likely to be non-adherent [61]. Therefore, these data show the need to screen for depression. Based on the results, the PHQ-2 was added as a screening tool for pharmacists to address and refer, if needed. The PHQ-2 is a commonly used 3 minute screening tool for major depression with a sensitivity of 83% and specificity of 92% [38].

A mixed methods approach, used for this study, is valuable in assessing and addressing processes affecting implementation of evidence-based interventions [62]. This methodology is a good framework for thinking about health services research that facilitated distinctive insights into multifaceted phenomena related to health care interventions, particularly with understudied populations such as older Hispanics [63]. This study is potentially beneficial because 1) we focus on a high-risk, understudied group (Hispanics with T2D) for whom culturally and linguistically appropriate medication adherence and disease management interventions have yet to be developed and tested and 2) we used a rigorous mixed methods technique to capture rich qualitative data that would serve to elucidate the participant responses from the quantitative questions depicting the challenges of Hispanics with T2D.

## Conclusion

Computerized tools to enhance the efficiency and effectiveness of appropriately educating and counseling patients may prove useful for pharmacists and other health care providers. From this pilot study, five dominant themes emerged from the participant interviews related to T2D management: 1) financial considerations, 2) polypharmacy, 3) social/family support, 4) access to medication/information, and 5) loneliness and sadness. The findings elucidate knowledge of how low-acculturated older Hispanics manage their T2D and how it impacts disease management and medication adherence. The themes that emerged and the additional comments from the clinical pharmacists and participants assisted in developing a computerized intervention. The goal of this intervention is to allow for a systematic and tailored approach to counseling. The ability for participants to answer questions throughout the intervention may allow health care providers to gather the necessary information to provide *precision counseling*. Findings from this study may help guide tailored interventions pharmacists can use to assist this patient population when providing CMM. Further studies are needed to determine health outcomes post intervention, such as increased HL and medication adherence in this study population.

## Supporting information

S1 File (DOCX)
