# Supplementary material for: Development of a computerized intervention to improve health literacy in older Hispanics with type 2 diabetes using a pharmacist supervised comprehensive medication management
Source: PLoS One. 2022 Feb 9;17(2):e0263264. doi: 10.1371/journal.pone.0263264 (PMC8827421; doi:10.1371/journal.pone.0263264)
Supplement: S1 File — (DOCX) [file pone.0263264.s001.docx]

Supplemental Information: Survey questions asked

Phase I

Participants (type 2 diabetes)

1. What is your age?
2. How do you gender identify?
3. What is your race?
4. What is your ethnicity?
5. What is your educational level?
6. How long have you had type 2 diabetes?
7. What was the most recent hemoglobin A1c level you remember?
8. What medications are you currently taking?
9. What comorbid conditions do you have besides type 2 diabetes?
10. How many medications for type 2 diabetes do you take and how does it affect the ability to remember taking them?
11. Does the schedule on how and when to take your medications affect the ability to take them?
12. Do you believe your medications for type 2 diabetes are helping you? (why or why not?)
13. How do you perceive your health?
14. Does your mood ever affect the way you take your medications? (If so, how?)
15. Does the cost of medications affect the way you take your medications? (If so, how?)
16. What type of social support do you have with taking your medications? Who helps you with taking medications?
17. Who do you ask or where do you go for information about medications?

*Spanish version*

1. ¿Quantos años tienes?
2. ¿Cómo identificas tu género?
3. ¿Cuál es su raza?
4. ¿Cuál es su etnia?
5. ¿Cuál es su nivel educativo (Hasta que grado fuistes)?
6. ¿Cuánto tiempo hace que tienes diabetes tipo 2?
7. ¿Cuál fue el nivel de hemoglobina A1c más reciente que usted recuerda?
8. ¿Qué medicamentos recien está tomando?
9. ¿Qué otras condiciones comórbidas tiene además de la diabetes tipo 2?
10. ¿Cuántos medicamentos para la diabetes tipo 2 toma y cómo eso afecta la capacidad de recordar haberlos tomado?
11. ¿El horario de cómo y cuándo tomar sus medicamentos le afecta como tomarlos?
12. ¿Cree que sus medicamentos para la diabetes tipo 2 le están ayudando? (¿por qué o por qué no?)
13. ¿Cómo percibe su salud?
14. ¿Alguna vez su estado de ánimo afecta la forma en que toma sus medicamentos? (¿Si es así, cómo?)
15. ¿El costo de los medicamentos afecta la forma en que los toma? (¿Si es así, cómo?)
16. ¿Qué tipo de apoyo social tienes para tomar sus medicamentos? ¿Quién le ayuda a tomar los medicamentos?
17. ¿A quién le pregunta o dónde va para obtener información sobre medicamentos?

Phase III

Participants (type 2 diabetes)

1. Repeated demographic questions 1-9 similar to Phase I.
2. Please think out loud as you look over the intervention. We want to know what you like and what you do not like. *After reviewing the intervention*, we asked the following:
   1. Are the modules for the intervention appropriately divided? If not, how would you break up the intervention?
   2. How long do you think each module would take to complete?
   3. How long do you wait in the office to see your doctor for type 2 diabetes?
   4. Overall, the computer program would be easy for you to use. (strongly disagree/disagree/agree/strongly agree)
   5. Learning to operate a touch screen computer for the intervention would be easy for you (strongly disagree/disagree/agree/strongly agree)

*Spanish version*

1. Repeated demographic questions 1-9 similar to Phase I.
2. Piense en voz alta mientras observa la intervención. Queremos saber lo que te gusta y lo que no te gusta. *After reviewing the intervention*, we asked the following:
   1. ¿Los módulos (sección) de la intervención están bien divididos? Si no, ¿cómo dividiría la intervención?
   2. ¿Cuánto tiempo crees que tomaría completer (terminar) cada modulo (sección)?
   3. ¿Cuánto tiempo espera el la oficina para ver a su médico por diabetes tipo 2?
   4. En general, el programa de computadora sería fácil de usar. (totalmente en desacuerdo/en desacuerdo/de acuerdo/totalmente de acuerdo)
   5. Aprender a operar una computadora con pantalla táctil para la intervención sería fácil para usted (totalmente en desacuerdo/en desacuerdo/de acuerdo/totalmente de acuerdo)

Participants (clinical pharmacists)

1. Repeated demographic questions 1-4 similar to Phase I.
2. What is your educational level? Please tell me what post graduate training you have completed (eg, residency/fellowship)? How many years have you practiced with this patient population?
3. Please think out loud as you look over the intervention. We want to know what you like and what you do not like. *After reviewing the intervention*, we asked the following:
   1. How would you break up the intervention in your practice?
   2. Anything you would revise, add, or delete?
   3. How long do patients usually wait in your practice?
   4. Overall, the computer program would be easy for patients to use. (strongly disagree/disagree/agree/strongly agree)
   5. Learning to operate a touch screen computer for the intervention would be easy for a patient (strongly disagree/disagree/agree/strongly agree)
   6. The intervention can save me time when conducting comprehensive medication management. (strongly disagree/disagree/agree/strongly agree)
   7. The intervention can allow me to systematically assess the needs of my patient. (strongly disagree/disagree/agree/strongly agree)
   8. The intervention would be easy to use in an outpatient practice when performing comprehensive medication management. (strongly disagree/disagree/agree/strongly agree)
